# Effect of Integrated Yoga Program Along With Pilates in Abdominal Hysterectomy Patients: A Case Report

**DOI:** 10.7759/cureus.46316

**Published:** 2023-10-01

**Authors:** Kratika R Dubey, Priyanka A Telang, Leksha Patel

**Affiliations:** 1 Community Health Physiotherapy, Ravi Nair Physiotherapy College, Datta Meghe Institute of Higher Education & Research (Deemed to be University), Sawangi, IND

**Keywords:** pelvic floor strength, physiotherapy, yoga, pilates, hystrectomy

## Abstract

Abdominal hysterectomy is a surgical procedure that involves the removal of the uterus through an incision in the abdominal wall. Hysterectomy is associated with a number of complications; hence, early mobilization and physiotherapy are necessary following surgery. This is a case report of a 45-year-old female who presented with complaints of abdominal pain associated with white discharge. Investigations showed an anterior submucosal fibroid measuring 3.2x2.7 cm and the patient underwent an abdominal hysterectomy. Early mobilization and physiotherapy were started on Day 6, which included breathing exercises, upper limb mobility, lower limb mobility, and positioning and postural advice. Yoga and pilates therapy were integrated into the protocol starting from the second week. The patient attended 30 days of therapy in two phases and was advised to continue even after discharge. We concluded that this unique approach of including pilates and yoga showed a positive impact on the patient's condition in terms of her quality of life, strength, endurance, and power, even decreasing her difficulty in performing activities of daily living.

## Introduction

Hysterectomy ranks second to cesarean section as the most often carried out surgical intervention during the reproductive age in various countries of the world. In order to treat a range of gynecological issues, it entails removing the uterine corpus entirely (total hysterectomy) or partially (subtotal or supracervical hysterectomy) [1]. An abdominal or vaginal approach was traditionally used to perform hysterectomies. Laparoscopic techniques have become more popular recently. Uterine cancer is one of the medical causes for hysterectomy, along with other non-cancerous uterine problems such as fibroids, endometriosis, prolapsed cervix, adenomyosis, and other uterine illnesses [2]. The rate of hysterectomy rates in India is 3.2%, with Andhra Pradesh having the highest rate (8.9%) and Assam having the lowest rate (0.9%). The incidence in India's rural areas is higher than in its cities. The majority of women undergo the operation in private institutions [2].

Numerous risks are associated with hysterectomy, including infection, venous thromboembolism, damage to the genitourinary and gastrointestinal tracts, bleeding, nerve damage, and vaginal cuff dehiscence [3]. To avoid all of these complications post surgery, the patient needs to begin physiotherapy as soon as possible. Postoperative physiotherapy includes encouraging them to get out of bed, enhancing their circulation, and preventing chest problems. By preventing or treating postoperative problems and providing physical rehabilitation to support the restoration of premorbid physical function, physiotherapy aids in the promotion of surgical recovery. Physiotherapy, while primarily focusing on physical recovery, may have an impact on a number of other categories. The treatment comprised pain management techniques, breathing exercises, pelvic muscle strengthening, and endurance training.

In this case report, our main focus is on yoga and Pilates along with conventional physiotherapy rehabilitation. Pilates is a form of physical training that makes use of resources like gravity and resistance to either help or hinder the execution of movements [4]. Pilates exercises are needed to enable the coordination of multiple muscle groups together, in contrast to typical resistance training in which muscles are treated independently [5]. Pilates workout regimen helps women's physical and psychological characteristics, including a 20% reduction in depression [6]. The Pilates technique emphasizes deep trunk stabilizing muscles in conjunction with pelvic floor muscles, as well as breathing. Exercises in the Pilates method are aimed at improving pelvic stability, mobility, and body alignment. Pelvic floor muscle exercises (PFME) are carried out in unison with breathing, and various positions of the trunk muscles are simultaneously recruited. These activities are crucial because breathing mechanics, maintaining intra-abdominal pressure, and continence all depend on one another [7]. Pilates helps to maintain or improve body mass index (BMI) because obesity has been found to be a significant risk factor following surgery, impairing cardiovascular and metabolic processes [8].

Yoga is an ancient practice that helps people find balance and health, and it has also been utilized as a therapeutic technique to ease the symptoms of many diseases [9]. It has been demonstrated to assist people in reducing their anxiety and stress. According to the study, yoga can be utilized in conjunction with standard postoperative treatment to benefit postoperative healing. Pain and psychological distress are common side effects of surgery for women who have gynecologic cancers suspected. Yoga could lessen these unfavorable surgical effects [10]. Yoga has numerous beneficial effects on quality of life and helps to improve a variety of symptoms, such as weakness, exhaustion, and mental impairments [11]. Along with lowering cancer symptoms and stress, it also aids in the prevention of cardiovascular disease, chronic pain, and persistent pain.

## Case presentation

A 45-year-old female, apparently healthy one year prior to presentation, started having pain in the abdomen (right iliac region), which was on and off, stabbing in nature and non-radiating. The pain was associated with a non-foul-smelling white discharge accompanied by itching. The patient also complained of irregular menstrual cycle and the flow needed three to four sanitary pads per day. Bowel and bladder were under control. She was gravida 2, para 2, with no history of abortion. The patient presented with the above-mentioned complaints at a private clinic where investigations showed some abnormality. For further management, she came to Acharya Vinoba Bhave Rural Hospital (AVBRH). The patient was a known case of hypertension for five years and was on hydrochlorothiazide (single dose a day) after breakfast. According to the Kuppuswami scale, the patient was upper middle class, lived in a "pucca" house on the ground floor, and used a Western type of toilet.

Investigations

Cytopathology examination showed an inflammatory smear with bacterial pathogens, but no malignant cells were found. Ultrasound showed that the uterus was retroflexed, of size 9x6.6x5 cm, and there was an anterior submucosal fibroid measuring 3.2x2.7 cm. The bilateral ovary appeared normal. No free fluid was noted in the peritoneal cavity.

Operative procedure

After obtaining consent, the patient was induced with spinal and epidural anesthesia. The abdomen was opened in layers. The rectus sheath was cut and muscle was separated. The uterus was identified and exteriorized. Bilateral round ligaments clamped, cut, and ligated. A window was created between the infundibulopelvic ligament and the ovarian ligament on the left. Retractors were placed into the incision. The bowel was packed with moist sponges and was gently dissected off the lower uterine segment and cervix. Uterine arteries were clamped, cut, and ligated bilaterally along with the uterosacral ligament. The uterus along with the cervix were removed. Vaginal vaults were sutured. The abdomen was closed in layers. Haemostasis was achieved and the patient was shifted to the ward.

Physiotherapy intervention

After the surgery, on postoperative Day 6, physiotherapy was started. In phase 1 (weeks 1-2), our main goal was to educate the patient regarding her condition, the importance of physiotherapy in this condition, and how early mobilization would help her in recovery. After discharge, physiotherapy was continued in the outpatient department. Proper in-bed positioning with the help of a pillow was taught to her. In the supine lying position, she was taught to use one pillow under the ankle, one pillow under the knee, and one pillow under the head for relaxation. Regular shifting of position helped in preventing secondary complications. Deep breathing exercises were taught to maintain oxygen saturation. The patient was advised to wear an abdominal splint and support the abdomen while coughing, sneezing, and using the toilet. Transcutaneous electrical nerve stimulator (TENS) of low frequency was applied on suitable points for 10-15 minutes to reduce postoperative pain. From the second week, the patient was encouraged to maintain an upright sitting position.

Pranayam was taught in which the patient was asked to inhale deeply through the left nostril while holding her right nostril closed with her right thumb. At its culmination, the nostrils were switched by closing off the left nostril and continuing to exhale smoothly through the right nostril. This was done for 10 repetitions, one set. After this, shoulder shrugs and scapular protraction and retraction were done along with the upper limb active range of motion. Active ankle-toe movement (10 repetitions, one set) was taught to maintain circulation. Active knee flexion and extension were given for lower limb mobility. From day 7-10, the patient's mobility was encouraged to increase independence. This included walking down the corridors or around her bed. This progressed to marching on the spot and heading to a chair from her bed. This raised the volume of the lungs, enhanced breathing, and decreased her hospital stay. The patient was taught Kegal's exercise, and she was asked to contract the pelvic muscles as if she were trying to hold the urine. In the third week, she was taught pelvic floor muscle exercise, Pilates (Table 1), and yoga to increase core muscle strength. All these poses were done for 10 repetitions, one set.

**Table 1:** Pilates exercises prescribed

Name and position of exercise	Description
Initial principals integration (supine)	Coordination of breathing, neutral supine, transverse abdominis, and pelvic floor muscle activation
Pelvic clock (supine)	Pelvic movement that mimics a clock causes the lumber spine to flex, extend, and rotate
Basic bridging (supine)	Knees in flexion and pelvic elevation
Adductor squeeze (supine)	Squeeze the adductor muscle between your knees while holding a ball
Bent knee fall out (supine)	a single inferior limb abduction with pelvic stabilization
Supine arm series (supine)	Arm workout in flexion, abduction, and rotation with trunk stabilization
Quadruped	Dissociation of one limb in quadrupedal stance with neutral spine.
Roll down series (seated)	Spine segmented flextion with band assistance.
Standing leg pump	Single-leg hip and knee flexion while supporting the trunk with both hands and both arms.
Assisted squads (standing)home exercise	Squats with a band to help stabilize the trunk

**Figure 1:**
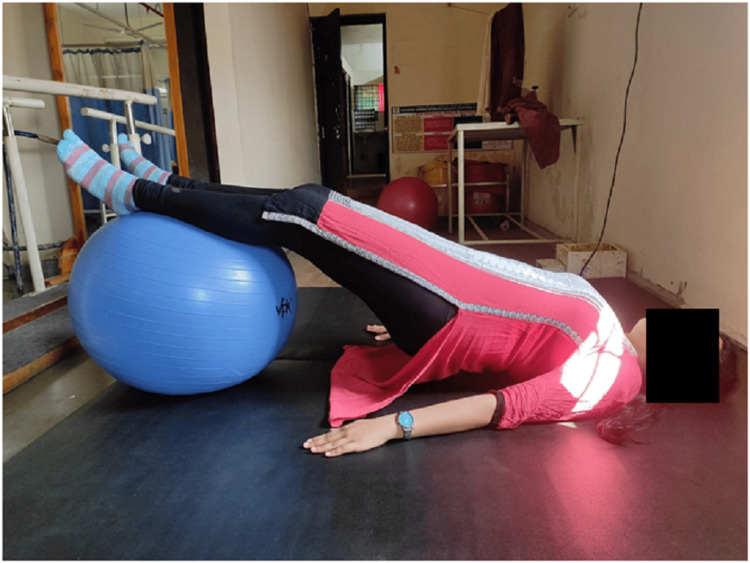
Pelvic bridging exercise

Figure 1 shows the pelvic floor exercise done with the help of a Swiss ball. In this, the patient was in a supine lying position, and placed a Swiss ball under their leg. Both the upper limbs were placed on each side over the plinth, and the patient was asked to lift the pelvis, and hold for five to seven seconds, and repeat 10 times. The contraction of abdominal muscles and back muscles against gravity improved the strength and endurance of the patient.

**Figure 2:**
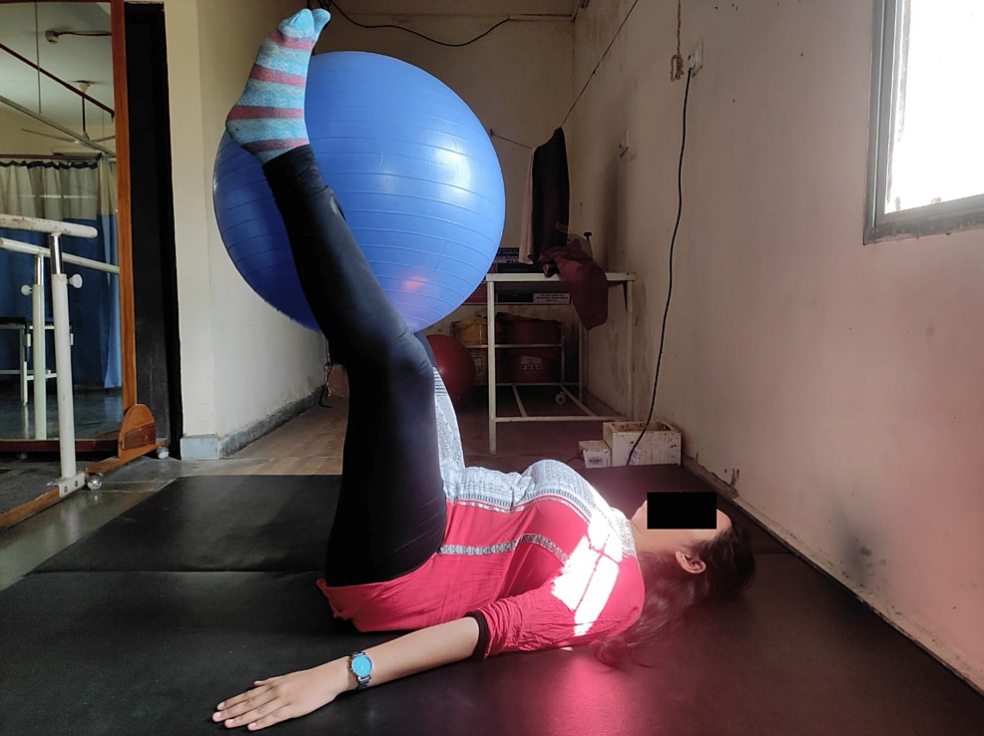
Core strength training with the help of physio ball

In week 4, our goal was to increase endurance and to increase the strength of the pelvic floor and abdominal muscles (Figure 2). All these poses were done for 10 repetitions, two sets. The patient was in a supine lying position and the Swiss ball was placed between the legs. She was then asked to lift it and contract the abdomen simultaneously. This helped strengthen the core, hip flexors, and hip adductors together. The patient was asked to take deep breaths continuously while performing yoga (Table 2).

**Table 2:** Yoga poses prescribed for core and back strengthening

Name of pose	Description
Bow pose	Improves stability and mobility. Flex the hip, extend knee, raise the trunk, and extend upper limb.
Cat-camel pose	Starting position is quadruped, sink the back towards the floor hold for 10 seconds, then arch the spine mimicking the camel hump.
Bird-dog pose	Strengthening abdominal and back muscles. Start from quadruped, extend right upper limb, and left lower limb simultaneously.
Boat pose	Benefits lower back and abdominal muscles. Also strengthens muscles of arm, thighs, and shoulder.

**Figure 3:**
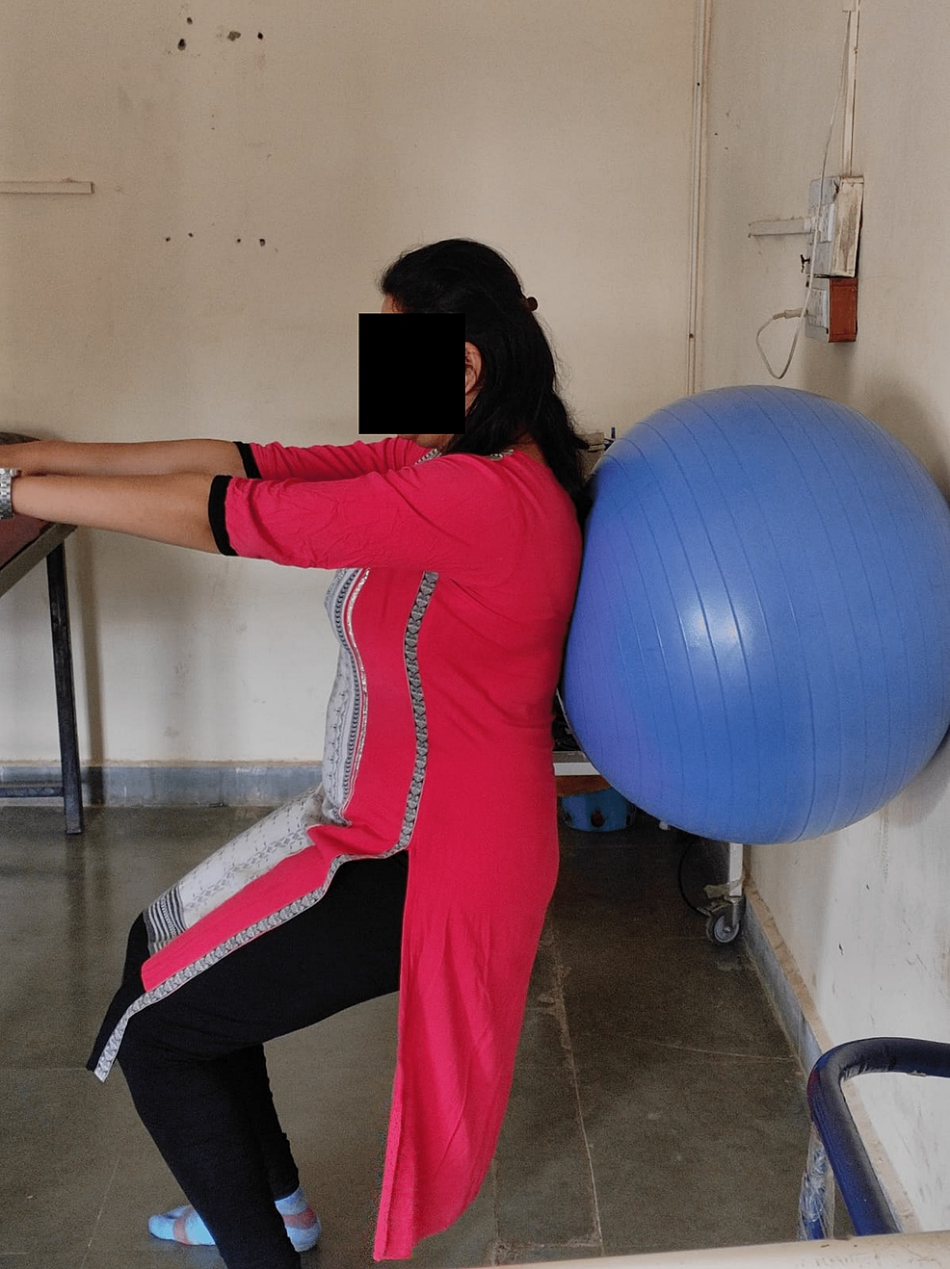
Strengthening with the help of physio ball

Figure 3 shows another exercise. The patient was in a standing position and the Swiss ball was placed between the wall and the patient's back. Both arms were flexed 90 degrees and the patient was asked to squat (bend the knees). This is a closed chain exercise that will help to improve posture by strengthening the quadriceps, gluteus maximus, paraspinal back muscles, pelvic floor muscles, and core abdominal muscles.

Naukasana, also known as boat pose, is very effective in strengthing core and back muscles. In this, the patient was in a supine position, shoulder flexed up to 90 degrees, with knees extended. The patient was asked to lift the head up to the scapula and the lower limbs had to be lifted together at the same time.

The results of the treatment protocol were recorded using various outcome measures (Table 3) such as a numerical pain rating scale, manual muscle testing, etc. 

**Table 3:** Outcome measures NPRS: Numerical Pain Rating Scale; MMT: Manual Muscle Testing

Outcome measures	Postoperative day 1	Day of discharge
NPRS	8/10	1/10
MMT of upper abdominal muscles	0 (no contraction)	Good
Barthel index	25/100	85/100
Pelvic floor impact questionnaire -7 (0-100 )	66.7	28.7

## Discussion

Even though hysterectomy has been the most popular non-obstetric surgical surgery for the past three decades, researchers have only just started to evaluate its results and numerous complications thoroughly. Despite a more extended hospital stay, more postoperative pain, a higher infection rate, and a slower return to normal activities, abdominal hysterectomy remains the most popular surgical procedure.

The ultimate objective of physical therapy rehabilitation is to promote independence and a return to normal, productive activities away from the hospital. In their study, Darware et al. found that physiotherapy treatment given immediately after gynecological surgery improves the patient's quality of life and that a planned exercise program benefits the patient more than traditional physiotherapy management and should be stressed to all patients after gynecological surgeries [12]. The findings of the study by Reddy and Frantz indicated that standard options for the postoperative care of patients following cesarean scar defect (CSD) and hysterectomy included mobilization, deep breathing exercises, and education [13]. The current case report focuses on an integrated approach of Pilates and yoga along with phase-wise physiotherapy protocol.

Gagnon et al. stated in 2005 that Pilates is a form of physical training that makes use of resources like gravity and resistance to either assist or resist movement execution [4]. These exercises, with a holistic approach, allow the synchronization of multiple muscle groups at once, in contrast to typical resistance training where muscles are worked independently [14]. Resistance training exercises have been found to have an impact on weight loss and maintenance [15]. Pilates exercises using the physio ball were shown to cause a reduction in obesity among females, improving glycemic control because weight gain following major gynecological surgeries results in low back pain, knee pain, and fatigue, increasing levels of dependence for daily functioning over time [16].

According to Fernandes et al., in the postoperative period of abdominal surgeries, respiratory physiotherapy is efficient in reversing atelectasis and in increasing oxygen saturation, and in their study, a respiratory exercise protocol was developed, resulting in the improvement of minute-volume and tidal volume [17]. Reddy et al. concluded that there is an increase in pulmonary function parameters like maximal voluntary ventilation (MVV)% and peak expiratory flow rate (PEFR)% in individuals after short-term yoga training (Pranayama). Thus the practice of yoga increases respiratory efficiency [18]. Sarang and Telles reported that a combination of yoga postures interspersed with relaxation improved measures of cardiopulmonary status in 40 male volunteers to a greater degree than relaxation alone [19].

Rao and his colleagues did a study on breast cancer patients undergoing surgery in which they concluded that yoga is an effective intervention for mood states, relieving symptoms, and improving the immune system [20]. Additionally, they noticed that the postoperative serum IgA levels were lower in the yoga group participants compared to controls and that CD56% decreased much less in the yoga group compared to the control group. However, the control group experienced a significant decline in CD4, CD8, and CD56 after surgery. The benefits of stress reduction and adaptability to the stress of surgery may be responsible for the alterations in lymphocyte subpopulations observed with yoga therapies. This may have promoted a decline in postoperative discomfort and a subsequent improvement in immunological outcomes.

Yoga's abilities to create stable autonomic balance, develop hypometabolic states, increase cardiopulmonary functions, improve immunological tolerance, develop neuroendocrine functions, improve emotionality, and create a calm state of mind to combat stress all contribute to its effectiveness in promoting health [20]. A study conducted on 30 geriatric women who participated in a 12-week Pilates training program showed that the Pilates exercise program improves physical and psychological aspects in women, including a 20% reduction in depression [6]. Hassan and Amin discovered that performing Pilate exercises boosted blood serotonin levels and decreased depression in women over the course of a 12-week research [21].

According to previous studies, a hysterectomy can lead to bladder changes [22]. Kegel exercises may be done to improve the strength of pelvic floor muscles and prevent urinary incontinence [23]. The main complaint of patients in the first week after surgery is postoperative pain; therefore TENS was used in areas associated with hysterectomy, and it reduced postoperative pain, demonstrating its efficacy as a non-drug alternative for this purpose. Studies indicate that TENS and acupressure are highly recommended for minimizing postoperative nausea and vomiting because of their positive effects [24].

## Conclusions

This case report showed that physiotherapy treatment carried out immediately after an abdominal hysterectomy helped to reduce hospital stay and prevent several secondary complications. In this whole intervention, a unique approach of including yoga with Pilates was used, and it proved to be effective as muscle strength, endurance, and quality of life improved. In addition to enhancing lung capacity and reducing respiratory complications, yoga practices like pranayam also had a calming and relaxing effect that helped the patient feel less anxious. Ideally, patients should continue this throughout their life and should avoid all high-impact exercises until at least 12 weeks post surgery.
